# Species‐range‐size distributions: Integrating the effects of speciation, transformation, and extinction

**DOI:** 10.1002/ece3.8341

**Published:** 2022-01-24

**Authors:** Nao Takashina, Michael J. Plank, Clinton N. Jenkins, Evan P. Economo

**Affiliations:** ^1^ Biodiversity and Biocomplexity Unit Okinawa Institute of Science and Technology Graduate University Onna‐son Japan; ^2^ Department of International Studies The University of Tokyo Kashiwa Japan; ^3^ School of Mathematics and Statistics and Te Pūnaha Matatini University of Canterbury Christchurch New Zealand; ^4^ Department of Earth and Environment Kimberly Green Latin American and Caribbean Center Florida International University Miami Florida USA

**Keywords:** diversification rate, geographic range‐size distribution, lineage‐through‐time plots, mathematical model

## Abstract

The species‐range size distribution is a product of speciation, transformation of range‐sizes, and extinction. Previous empirical studies showed that it has a left‐skewed lognormal‐like distribution. We developed a new mathematical framework to study species‐range‐size distributions, one in which allopatric speciation, transformation of range size, and the extinction process are explicitly integrated. The approach, which we call the gain‐loss‐allopatric speciation model, allows us to explore the effects of various speciation scenarios. Our model captures key dynamics thought to lead to known range‐size distributions. We also fitted the model to empirical range‐size distributions of birds, mammals, and beetles. Since geographic range dynamics are linked to speciation and extinction, our model provides predictions for the dynamics of species richness. When a species‐range‐size distribution initially evolves away from the range sizes at which the likelihood of speciation is low, it tends to cause diversification slowdown even in the absence of (bio)diversity dependence in speciation rate. Using the mathematical model developed here, we give a potential explanation for how observed range‐size distributions emerge from range‐size dynamics. Although the framework presented is minimalistic, it provides a starting point for examining hypotheses based on more complex mechanisms.

## Graphical abstract

We developed a new mathematical framework to study species‐range‐size distributions, one in which allopatric speciation, transformation of range size, and the extinction process are explicitly integrated. Our model captures key dynamics thought to lead to known range‐size distributions. We also fitted the model to empirical range‐size distributions of birds, mammals, and beetles.

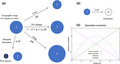

## 1 INTRODUCTION

The distribution of species‐range sizes is a key pattern of interest in macroecology, providing a basic spatial profile of the earth's biodiversity (Gaston, 1998). Despite the broad range of biological dynamics that potentially influence such a macroscopic pattern, a relatively consistent form is often observed across taxa and regions: Species‐range sizes have a lognormal distribution with left skew (Brown et al., 1996; Gaston, 1998; Gaston & Blackburn, 1996, 2008; Gaston & He, 2002; Noonan, 1990; Orme et al., 2006; Ruggiero, 1994). Gaston (1998) provides skewness values from a wide range of taxonomic groups where 19 out of 22 range‐size distributions have a skewness between −1.26 and −0.03 on a log‐axis (note right‐skewed distributions were also reported but these were minor).

This generality in the form of the pattern raises the possibility of some similarly general mechanism. While there is a long list of processes that potentially influence range‐size distributions, the shapes of these distributions are ultimately a product of speciation, extinction, and transformation of the range (Gaston, 1998). Therefore, exploring these patterns leads to a central question in ecology and biogeography: How do the processes of speciation, extinction, and transformation mutually interact to shape range‐size distributions? (Brown et al., 1996; Gaston, 1998).

Given the large spatial and temporal scales involved, quantitative approaches have been central to the study of species ranges. These include studies with a particular focus on inferring speciation modes (Cardillo, 2015; Phillimore & Price, 2008; Skeels & Cardillo, 2019), phylogenesis (Albert et al., 2017; Pigot et al., 2010), niche evolution (Rangel et al., 2007, 2018), size‐age relationship of geographic ranges (Pigot et al., 2012), and the heritability of range size (Borregaard et al., 2012). In addition to the focus on individual species‐range sizes, previous studies also shed light on the shape and the underlying mechanisms of the emergent distributions of range size across many species (e.g., Alzate et al., 2019; Anderson, 1985; Gaston & He, 2002; Pigot et al., 2010; Rangel et al., 2007). In early work, Anderson (1985) focused on range‐size distributions using an algorithm with nine different scenarios for range dynamics (speciation is associated with an extinction event in the algorithms) to generate range‐size distributions resembling faunal data for North American vertebrates. Later, Gaston & He (2002) developed a stochastic model that describes range‐size dynamics driven by population dynamics of a single species, and they showed that the generalized equilibrium range‐size distributions produced by the model fit well to range‐size distributions of several taxonomic assemblages. Pigot et al. (2010) built a spatially explicit numerical algorithm to simulate geographic range evolution where new species arise via vicariance or peripatry, and they reproduced empirically observed range‐size distributions across bird genera that lead to diversification slowdown in the reconstructed phylogenetic tree.

While these efforts have been illuminating, and detailed spatially explicit algorithms are available (e.g., Rangel et al., 2018), we still lack a comprehensive understanding of the assumptions necessary and sufficient to realize observed patterns of range‐size distributions. For example, we do not have concrete insight into the mutual importance of key processes, range transformation, extinction, and speciation (Gaston, 1998). Also, there are contrasting views regarding the effect of range size on speciation rate (Gaston, 1998; Jablonski & Roy, 2003; Rosenzweig, 1995; Tokeshi, 1996), and the effect of speciation rates combined with other key processes on emergent range‐size distributions has not yet been well explored.

We develop a new class of macroecological model describing key processes of range‐size dynamics that we call the *gain‐loss‐allopatric speciation* (GLAS) model. The GLAS model accounts for the key processes shaping ranges: the gain in area through dispersal, the loss of area due to local extinction, and the splitting of a single range into multiple ranges through allopatric speciation. Our mathematical framework is simple and flexible to accommodate various assumptions, and we examine several scenarios of allopatric speciation. While *a priori* we could expect any number of range‐size distributions to be realized, we ask whether the fact that ranges are subject to gain, loss, and speciation dynamics places any constraints on their distributions. While in principle such a modeling approach could become very complex when incorporating a broad range of pertinent processes occurring in a region (e.g., Rangel et al., 2018), we take a minimalist approach to address general cases. We do not deal explicitly with species interactions (as, e.g., Gaston & He, 2002; Pigot et al., 2010), the genetic mechanisms of speciation, environmental heterogeneity, or change, or any number of other potential elaborations. Rather, we model range‐size distributions in terms of phenomenological rates of range growth, contraction, and speciation, which are implicitly affected by all those mechanisms.

The dynamics of species‐range‐size distributions can also affect the diversification process and structure of the associated phylogeny (Albert et al., 2017; Pigot et al., 2010). If speciation and extinction are linked to range size, then lineage diversification rates can be affected by dynamical changes in the range‐size distribution itself. This can occur by concentrating species at sizes with lower or higher speciation and extinction rates. Hence, investigating this theoretically provides an opportunity to link range dynamics to macroevolutionary processes. The dynamics of species diversity are also a central focus in macroecological studies (Nee, 2006; Nee et al., 1992; Rabosky, 2009). Diversification slowdowns, often discussed via lineage‐through‐time plots (Harvey et al., 1994; Nee et al., 1995), are a common feature of inferred lineage dynamics (Cusimano & Renner, 2010; Moen & Morlon, 2014; Morlon et al., 2010; Nee, 2006; Yoder et al., 2010), yet biological explanations for the underlying mechanisms (e.g., diversity‐dependent speciation) are still under active investigation (Condamine et al., 2019; Moen & Morlon, 2014; Pannetier et al., 2021). We demonstrate that the GLAS model provides an opportunity to examine the effect of range‐size dynamics on the diversification rate.

We find that left‐skewed distributions on a log‐axis are predicted under various speciation scenarios and parameter sets. The left‐skewed distributions are realized by the similar rates of the expansion and contraction of geographic ranges with the moderate supply of new species. We demonstrate that these three parameters regulate the shape of emergent range‐size distributions. Interestingly, our model can generate diversification slowdown in the dynamics of the number of species under all speciation scenarios investigated, even though the model has neither a diversity dependence effect nor a species interactions.

## 2 MATERIALS AND METHODS

### 2.1 Gain‐loss‐allopatric speciation model

Here, we give an overview of the gain‐loss‐allopatric speciation (GLAS) model. See Appendix 1 for complete discussion of the model. The GLAS model contains minimal but essential factors to describe range‐size dynamics and to recover empirical range‐size distribution: allopatric speciation, extinction, and transformation of the range size (Gaston, 1998).

**FIGURE 1:**
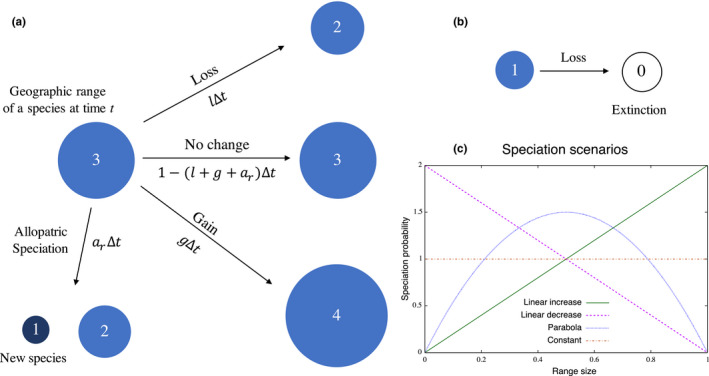
Schematic diagram of the model. Each species has a geographic range size at given time *t* that is labeled by a number. (a) Within a small time period Δt, a species range can increase in size, decrease in size, or undergo allopatric speciation leading to two smaller range sizes with one new species. (b) Extinction can occur if a species has the smallest allowable range size (labeled 1 in this example) and then decreases in size. (c) Four scenarios for the dependence of speciation rate on normalized range size (0≤x≤1). The probability distribution is described by a beta distribution and the parameter values for each scenario are *Linear increase*: α=2, β=1; *Linear decrease*: α=1, β=2; *Parabola*: α=2, β=2; and *Constant*: α=1, β=1. See the main text for more details

Let us assume that a species has a geographic range size *r* at time *t*, and experiences gain in its range size by one unit (r→r+1) or loss by one unit (r→r‐1) at rates *g* and *l*, respectively. In addition, allopatric speciation can occur at a size‐dependent rate *a_r_*, causing the subdivision of a geographic range to produce two smaller range sizes *r*_1_ and *r*_2_ with one new species (Figure 1a). We assume for simplicity that the sum of the two subdivided range sizes is equivalent to the parent range size $r_{1}+r_{2}=r$, and all combinations of split size are equally likely. We define an extinction as the event that a species reaches range size 0, and it only occurs from *r* = 1 (Figure 1b). We assume that each species‐range size changes independently of other species. These assumptions are translated into the following stochastic process that describes the dynamics of P(r,t), the expected number of species with range size *r* at time *t*:

(1) $$\frac{dP\left(r,t\right)}{\mathrm{dt}}=gP\left(r‐1,t\right)+lP\left(r+1,t\right)+2\sum_{r^{\prime }>r}\frac{a_{r^{\prime }}}{r^{\prime }‐1}P\left(r^{\prime },t\right)‐\left(g+l+a_{r}\right)P\left(r,t\right).$$

Equation (1) describes the dynamics of geographic range‐size distribution over time. It is a class of continuous‐time random walk (Gardiner, 2009; Karlin & Taylor, 2012) with an additional term for the effect of allopatric speciation.

It is worth noting that we assume that individual processes within each geographic range induce change of geographic range by “one unit,” and hence, the size of change is a constant regardless of the geographic range size. However, it may also be sensible to assume that environmental factors cause the size shift, and lead to a size‐dependent rate of change. We will discuss this effect in the Discussion and provide an example.

Because the GLAS model describes speciation and extinction events, it also generates dynamics of the total number of species, an exponential increase, decrease, or stable species number depending on the value of the dominant eigenvalue (see Appendix 1 for details and an example of eigenvalues provided in Figure A1).

### 2.2 Continuum limit of the GLAS model

It is reasonable to consider that the geographic range size changes continuously rather than in a discrete manner. When the step size of the range size is small (r∼r+1) in Equation (1), we introduce a new variable u(x,t) where the number of species with range size between *x* and x+Δx at time *t* is described by u(x,t)Δx. Then, we obtain the following integro‐differential equation that describes the dynamics of the number of species u(x,t) with range size *x* at time *t* (See Appendix):

(2) $$\frac{\partial u\left(x,t\right)}{\partial t}=‐V\frac{\partial u\left(x,t\right)}{\partial x}+D\frac{\partial^{2}u\left(x,t\right)}{\partial x^{2}}+2\int_{x}^{1}\frac{a_{x^{\prime }}}{x^{\prime }}u\left(x^{\prime },t\right)dx^{\prime }‐a_{x}u\left(x,t\right),$$

where V=(g‐l)Δx and $D=\left(g+l\right)\Delta x^{2}/2$. Note the assumption that the ancestral range is divided into two ranges with an equal probability of any split size, and so yields the average split proportion 25:75. Possibility of split asymmetry in marine mollusks was suggested in Pigot et al. (2012). Also, Budding speciation, by which a reproductively isolated smaller‐ranged species is originated from a larger‐ranged species by a highly asymmetric fashion, may be common in plant species (Anacker & Strauss, 2014; Grossenbacher et al., 2014), and our model can capture this speciation mode as this is a potentially (spatially overlapped) subdivision of a geographic range. Here, without loss of generality, we set the range size to lie in the interval 0≤x≤1, with 1 being the size of the entire domain (the maximum possible range, e.g., the size of a continent). Assuming that an extinction event occurs only when a range size reaches *x* = 0 and no species exits the domain from the upper bound *x* = 1 (i.e., no‐flux condition), we need mixed boundary conditions. Namely, a Dirichlet boundary condition for the lower boundary u(0,t)=0 and the Robin boundary condition for the upper boundary ‐Vu(1,t)+D∂u(1,t)/∂x=0. We can derive an analytical solution of Equation (2) when speciation increases linearly with range size by imposing extra conditions. However, the analytical form is rather complicated and computationally expensive due to an infinite summation. For the reader's convenience, we provide the derivation in Appendix 2. In the following, we will perform numerical analysis of Equation (2). Also, we will focus on deterministic characteristics of the GLAS model to directly discuss mutual importance of speciation, transformation, and extinction events, while Equation (1) allows us to investigate stochastic processes.

### 2.3 Scenarios of allopatric speciation

To complete the model Equations (1) and (2), we need to define the size dependency of speciation rate *a_x_*. However, as discussed in the Introduction, this is rather contentious. We assume four potential scenarios to capture some of the existing hypotheses, where the probability of speciation: (i) increases with range size (Rosenzweig, 1995; Tokeshi, 1996); (ii) decreases with range size (Jablonski & Roy, 2003); (iii) peaks at an intermediate size, which is attributed to a hypothesis that a high dispersal ability can lead to large ranges and inhibit speciation (e.g., Claramunt et al., 2012; Gaston, 1998; Mayr, 1963); or (iv) is independent of range size. We examine how these four speciation scenarios affect the form of the range‐size distribution. We use the beta distribution to model these four probability distributions with a single probability distribution function (PDF). The PDF *a_x_* has the form $a_{x}=ax^{\alpha ‐1}{\left(1‐x\right)}^{\beta ‐1}/B\left(\alpha ,\beta \right)$, where *a* is the underlying speciation rate and B(α,β) is the beta function. We use the simplest possible form to represent the above‐mentioned four scenarios: (i) *Linear increase*: α=2, β=1($a_{x}=2ax$); (ii) *Linear decrease*: α=1, β=2 ($a_{x}=2a\;\left(1‐x\right)$); (iii) *Parabola*: α=2, β=2 ($a_{x}=6ax\;\left(1‐x\right)$); and (iv) *Constant*: α=1, β=1 ($a_{x}=a$). The probability distributions of these speciation models are shown in Figure 1c. We also discuss nonlinear cases in Appendix.

### 2.4 Summary of situations investigated

#### 2.4.1 Range‐size distributions

Having completed Equation (2) with a speciation scenario, we can compute the range‐size distributions and the dynamics of the total species number over time. To facilitate comparisons between different speciation scenarios, we will present range‐size distributions scaled by the maximum possible range size (i.e., area of the domain) where all species‐range sizes span the range 0≤x≤1. To quantify the range‐size distribution, we calculate skewness and kurtosis statistics, which provide location‐ and scale‐free descriptors of distributions (e.g., normal distribution gives 0 values for both measures). Since the range size is scaled by the area of the domain, the relative values of parameters are important rather than their absolute values. Without loss of generality, we scale the gain rate and speciation rate by the loss rate, allowing us to set the loss rate l=1. This is a convenient approach to investigate the relative importance of multiple factors affecting range size. Also, this facilitates the investigation of range‐size distributions of two largely different taxonomic groups whose parameter values are of different order. Note since the model is linear, scaling each rate by a constant value gives the same equilibrium range‐size distribution.

Although we use a normalized model to investigate range‐size distributions across different taxonomic groups, this approach does not lose any qualitative property of the original model. The same analysis could be applied for any specific case with empirically estimated parameter values. Within these settings, we examine multiple sets of gain rates *g*, including the case g>l, g=l, and g<l, and multiple orders of the underlying speciation rates including the case where there is no speciation a=0. We also perform model fitting to data for mammals and birds in the Americas, and *Harpalus* carabids in North America north of Mexico. Since empirically observed range‐size distributions often span multiple orders of magnitude, we employ the logarithmically transformed GLAS model (Equation A13) to compute the range‐size distribution in the analysis where all the characteristics remain unchanged (see Appendix for details).

#### 2.4.2 Species number

Since the GLAS model characterizes speciation and extinction events, it produces the dynamics of total number of species $N\left(t\right)=\int_{0}^{1}u\left(x,t\right)\;dx$ where the number of species is also a continuous value as u(x,t) is a continuous value. Given such dynamics, it is possible to calculate diversification rate λ(t) over time, the difference between the speciation rate at time *t* and extinction rate at time *t*. Since the diversification rate has the following relationship λ(t)=dlogN(t)/dt and the extinction rate is calculated by $Du^{\prime }\left(0,t\right)/\int_{0}^{1}u\left(x,t\right)dx$ (Appendix 2), it is possible to compute all these time‐dependent rates along with the lineage‐through‐time plot. Along with these continuous representations, we also provide the statistic *r* (Etienne & Rosindell, 2012; Pigot et al., 2010) where *r* < 0 and *r* > 0 indicate a slowdown and speedup of the diversification rate, respectively, over the period of calculations.

### 2.5 Species‐range data

Species‐range data for birds (*n* = 365) and mammals (*n* = 628) in North America north of Mexico were from BirdLife International (BirdLife International & Handbook of the Birds of the World, 2017) and the IUCN Red List (IUCN, 2017) databases. Range size was calculated in the Eckert IV equal‐area projection. We excluded seabirds as our focus was species in continental areas. We excluded parts of species ranges labeled as introduced or vagrant. For birds, we used only the breeding range. Data for *Harpalus* carabids (*n* = 54) in North America north of Mexico are from Noonan (1990) where the range size was calculated based on occurrence data.

We used 17,683,892 km^2^ to normalize the range sizes (i.e., to organize all the range sizes between 0 and 1). Note the choice of the size for the normalization does not affect the shape of range‐size distributions (e.g., variance, skewness, and kurtosis), but it merely causes a shift on the *x*‐axis.

### 2.6 Model fitting

Although we do not have an explicit form of the likelihood function with arbitrary parameters for speciation scenarios *α* and *β* of Equation (2), we can perform data fitting via simulated annealing, an optimization algorithm, based on a log‐likelihood using a numerically calculated range‐size distribution. Detailed settings of the optimization algorithm are found in Appendix 3. In this process, we set the range of speciation parameters *α*, *β* ∈ [1,50]. We sampled the range‐size distribution when the changes in skewness and kurtosis between time steps become smaller than 10^−5^ (i.e., convergence to equilibrium) and eliminated parameter sets which cause species number smaller than 0.9 until the convergence. Note the value to judge the numerical convergence is different from the value used in other numerical simulations to reduce the simulation time of the optimization algorithm. Since we are interested in relative significance of gain, loss, and speciation, we again set the loss rate to 1.

## 3 RESULTS

### 3.1 Model predictions

Given a speciation scenario, and the three factors affecting range‐size dynamics, gain rate *g*, loss rate *l*, and an underlying allopatric speciation rate *a*, we can numerically solve the GLAS model. We observed that the range‐size distribution approaches a unique equilibrium distribution regardless of initial conditions after a certain simulation period. We sampled the range‐size distribution when the absolute difference in skewness (kurtosis) at two arbitrary time points (we sampled every 1000 time steps for numerical convenience) became smaller than 10^−7^. Figure 1a shows example dynamics of the range‐size distribution under the scenario of linearly increasing probability of speciation with range size and underlying speciation rate *a* = 0.1. Starting with a single species with a range size 0.1, the range‐size distribution converges to an equilibrium distribution that is comparable to some existing data: a left‐skewed curve on a log‐scale (Brown et al., 1996; Gaston, 1998; Gaston & Blackburn, 1996, 2008; Gaston & He, 2002; Noonan, 1990; Orme et al., 2006; Ruggiero, 1994).

**FIGURE 2:**
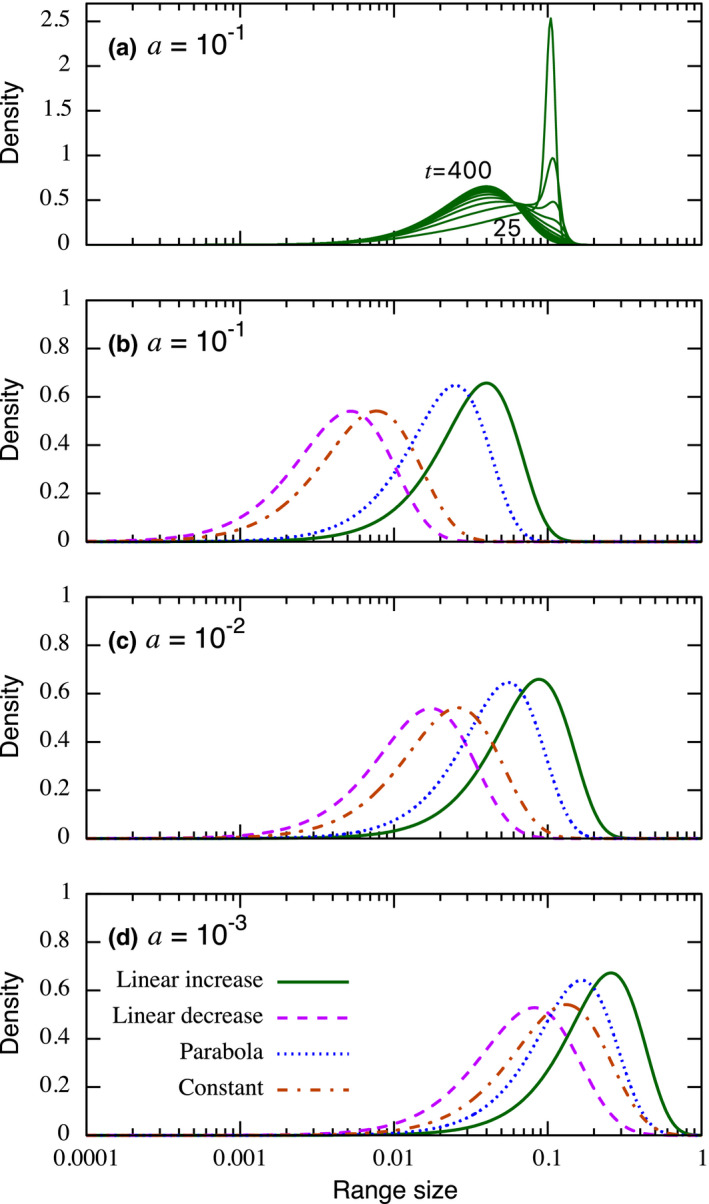
Normalized range‐size distributions that typically show a lognormal feature but are left skewed. (a) The time evolution of a range‐size distribution starting with a single species with range size 0.1 when the speciation scenario is linear increase. The range‐size distribution is recorded every 25 time units for visualization until t=400. (b) Equilibrium range‐size distributions with four speciation scenarios. Other parameter values used are l=1, g=1.2 for (a) and (b), and g=1.1 for (c) and (d). Also, parameters for the speciation scenarios (*α*, *β*) are (2, 1) (linear increase); (1, 2) (linear increase); (2, 2) (parabola); and (1, 1) (constant)

Left‐skewed range‐size distributions are observed for the other speciation scenarios, although distributions tend to shift to the left‐side on the *x*‐axis for the linear decrease and constant speciation scenarios (Figure 2b). Left‐skewed distributions are also observed when changing the speciation rate across multiple orders of magnitude (Figure 2c,d), although its magnitude of influence on the shape of the range‐size distribution is relatively small compared with the effect of alternative speciation scenarios (Table 1). However, a smaller underlying speciation rate causes a right shift of the distribution, since it suppresses the chance of allopatric speciation, which is the mechanism that pulls the distribution toward the left. As a result, the distributions of the linear decrease and constant speciation scenarios come closer to the other scenarios. We provide heat maps of skewness and kurtosis of the species‐range‐size distributions across parameters in Figures A2 and A3 showing the robustness of these summary parameters provided by the left‐skewed distribution.

**TABLE 1:** Summary of (skewness, kurtosis) from each curve in Figure 2

*a*	Speciation scenarios			
	Linear increase	Linear decrease	Parabola	Constant
10^−1^	(−1.016,1.913)	(−0.777,1.182)	(−0.996,1.847)	(−0.780,1.187)
10^−2^	(−1.020,1.926)	(−0.771,1.170)	(−0.989,1.832)	(−0.780,1.187)
10^−3^	(−1.045,2.013)	(−0.732,1.100)	(−0.953,1.783)	(−0.780,1.187)

If the underlying speciation rate is zero or sufficiently small, the mechanism to reduce the range size by supplying a new species is suppressed and the number of species declines over time (if an initial population size is larger than 1). As a result, the left skew vanishes and many species arrive at the largest possible range size when g≥l (Figure A4) and this corresponds to the left‐top region of each panel in Figures A2 and A3. Figures A2 and A3 also show that a similar result occurs when the gain rate is substantially larger than the loss rate and the underlying speciation rate, since this also suppresses the mechanism to reduce the range sizes.

The left‐skewed range‐size distribution is widely observed across the parameter space, including the case of nonlinear speciation scenarios (Figure A5), as long as there is a mechanism that avoids many species growing to the maximum possible range size. Although the gain rate tends to be larger than the loss rate (g>l) to have a positive species growth rate, it can still show a left‐skewed distribution even when the species growth rate is negative (Figure A6) and when speciation rate is zero and with a smaller gain rate than the loss rate (g<l; Figure A4) in the course of all species extinction.

**FIGURE 3:**
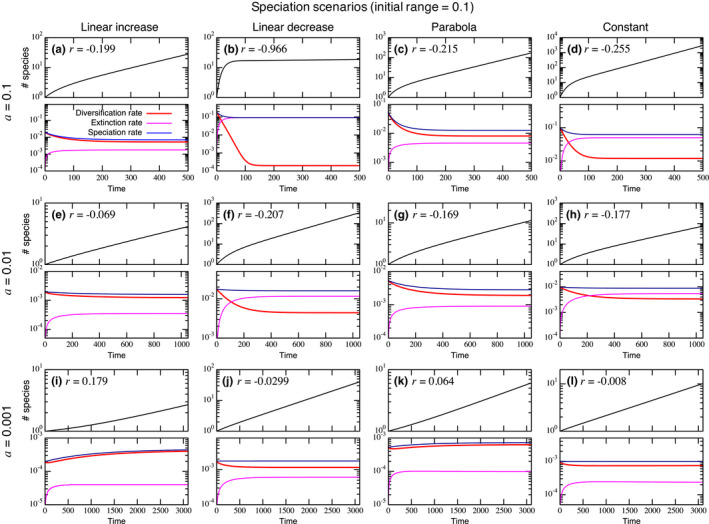
In each panel, (top) lineage‐through‐time plot and the associated statistic *r*, and (bottom) diversification, extinction, and realized speciation rates. (a)–(l) correspond to the scenarios shown in Figure 2. Each simulation is started with a single species with a range size 0.1. Each column shows a different speciation scenario (left to right: linear increase, linear decrease, parabola, and constant), and each row represents a different underlying speciation rate (top: *a* = 0.1; middle: *a* = 0.01; and bottom *a* = 0.001). Other parameter values used are l=1 and g=1.2 for (a–d) and g=1.1 for the rest

The GLAS model characterizes speciation and extinction events, and it produces the dynamics of total number of species. Starting with a single species, we found the diversification rate can decrease, without a diversity‐dependent effect, as it approaches the equilibrium diversification rate (Figure 3). Typically, diversification slowdown is associated with decline of the realized speciation rate and increase in the extinction rate as it approaches an equilibrium. When the speciation rate increases over time, slowdown is less likely to occur but it is still possible if the extinction rate also increases fast enough to suppress the effect of speciation as in Figure A8f. Speciation scenarios also affect these dynamics. The linear increase scenario tends to require larger initial range size to show the slowdown, while the linear decrease still can show the strong slowdown with a smaller range size. This trend can be explained by the match between the initial range size and the possibility of speciation. Namely, if an initial range has larger likelihood for speciation, it can produce a higher accumulation of species at an initial phase than the range size with lower likelihood of speciation. We provide these numerical results with two other initial range sizes (0.05 and 0.5) for each combination of the three underlying speciation rates (10^−1^, 10^−2^, and 10^−3^) with four speciation scenarios (Figures A7 and A8) and three nonlinear speciation scenarios with initial range sizes (0.05, 0.1, and 0.5; Figures A9, A10, A11). For nonlinear speciation scenarios, similar discussions are possible except for some situations. For example, the slowdown can occur when both speciation and extinction show a decreasing trend (Figure A11a,c) that is not observed with linear speciation scenarios. Also, nonlinear increasing and decreasing speciation scenarios generally induce more significant differences compared to these linear counterparts.

### 3.2 Model fits to empirical data

**FIGURE 4:**
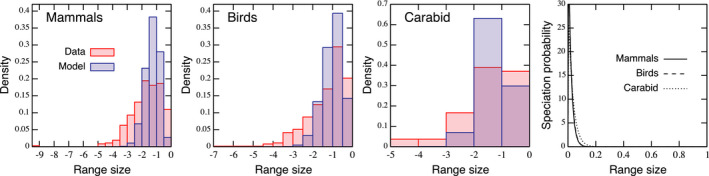
Model fit to the datasets for mammals (*n* = 365), birds (*n* = 628), and *Harpalus* carabids (*n* = 54) in North America north of Mexico (left three panels), and estimated speciation scenarios (right panel). The estimated parameters are in Table 2

**FIGURE A1:**
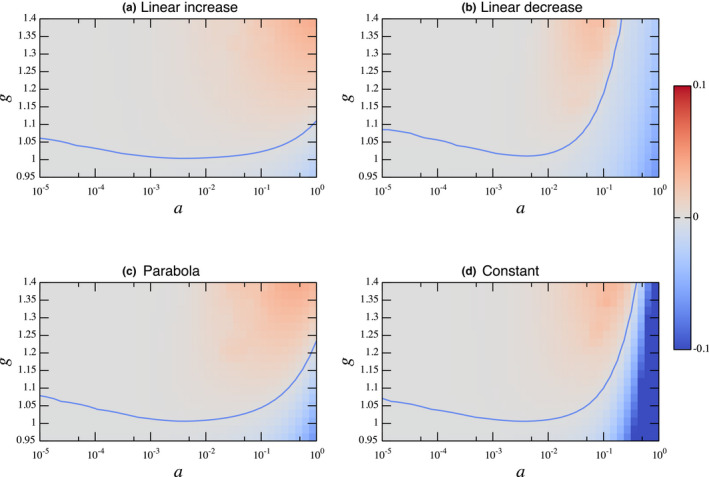
Dominant eigenvalues Re(λmax) across parameters with four speciation scenarios (a) constant, (b) linear increase, (c) linear decrease, and (d) parabola with range size in the case of the normalized range size (0≤x≤1). The curve in each panel corresponds Re(λmax)=0, and it divides the region into Re(λmax)>0 (upper) and Re(λmax)<0 (lower), corresponding to species increase or decrease, respectively

**FIGURE A2:**
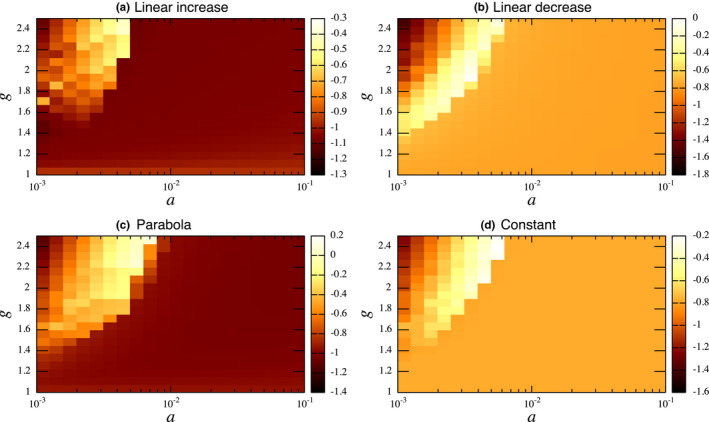
Heat map of skewness of the normalized range‐size distribution under four speciation scenarios. The left‐top region in each panel tends to show the species accumulation at the largest possible range size. The loss rate is l=1. Parameters for the speciation scenarios (*α*, *β*) are (2, 1) (linear increase); (1, 2) (linear increase); (2, 2) (parabola); and (1, 1) (constant)

**FIGURE A3:**
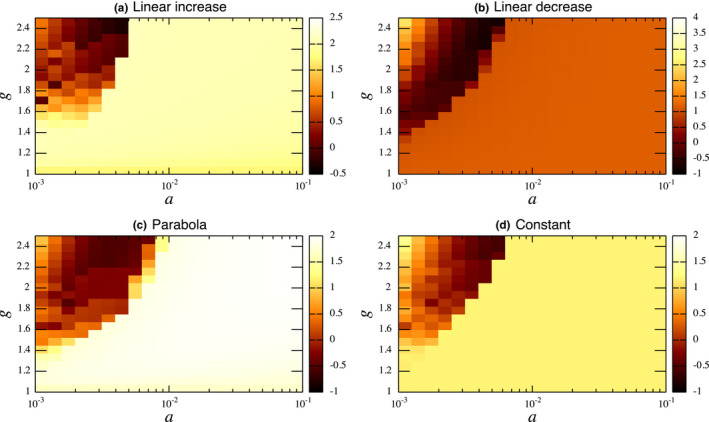
Heat map of kurtosis of the normalized range‐size distribution under four speciation scenarios. The left‐top region in each panel tends to show the species accumulation at the largest possible range size. The loss rate is l=1. Parameters for the speciation scenarios (*α*, *β*) are (2, 1) (linear increase); (1, 2) (linear increase); (2, 2) (parabola); and (1, 1) (constant).

Figure 4 shows the empirical and fitted histograms and shape of estimated speciation scenarios. The estimated parameters and values of skewness and kurtosis are summarized in Table 2. We found that our model tends to show narrower equilibrium distributions (spanning around 3 orders of magnitude) than the empirical datasets (5 or more orders of magnitudes). The speciation scenarios selected are nonlinear‐decreasing functions, but the parameter values are close to the domain boundaries of parameter space defined. Widening parameter spaces for speciation scenarios (i.e., *α* and *β*) allows more extreme, and perhaps unrealistic, speciation scenarios and may not improve prediction.

**TABLE 2:** Estimated parameters by the model fitting in Figure 4

Taxon	*g*	*a*	*α*	*β*	Skewness (data), kurtosis (data)
Mammals	1.65	0.0013	1.01	50.00	−0.504 (−0.679), 0.166 (0.0744)
Birds	5.33	0.0062	1.08	50.00	−0.618 (−0.748), 0.244 (0.749)
*Harpalus* carabids	1.10	0.0004	1.03	30.97	−0.387 (−1.136), 0.104 (0.698)

This casts a limitation of the model fit to a continental scale dataset with wide taxonomic scales. However, this may not be surprising as the model is minimalistic and assuming homogeneous biological parameters between species. In fact, our model leads to a better fit to the carabid dataset, which shows narrower distribution due to narrower taxonomic scales in the dataset. Also, the limitation in the model fits does not reduce the model utility because the left‐skewed distributions are observed at various taxonomic scales. Our model shows that homogeneous parameters are sufficient to recover this characteristic rather than the shape is a product of high heterogeneity in biological parameters. A wider distribution range may be the product of aggregating heterogeneous components from various taxonomic groups. Our model provides a better understanding at the level of such a component.

## 4 DISCUSSION

### 4.1 Left‐skewed range‐size distributions

**FIGURE A4:**
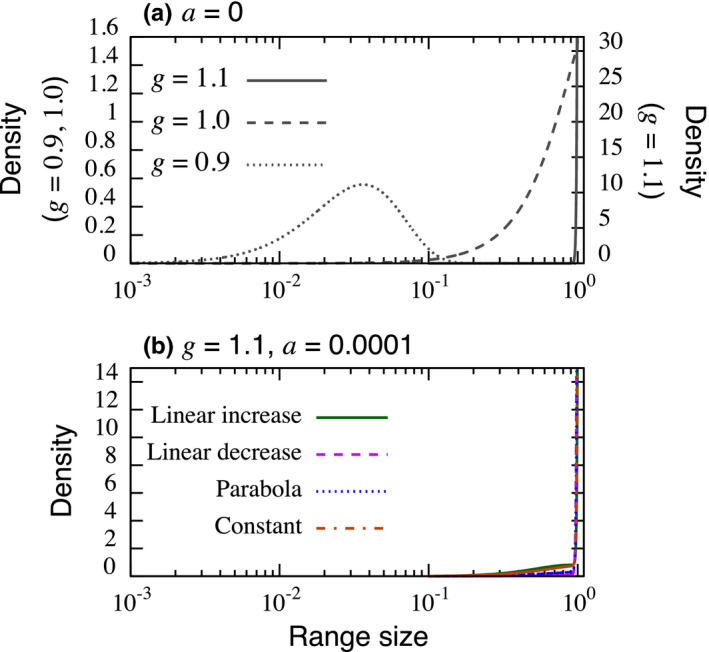
Normalized range‐size distributions with (a) no speciation and (b) a=0.0001. All scenarios cause a negative growth rate of the number of species. We sampled the range‐size distribution when either species number becomes 0.01 or range‐size distribution converged to equilibrium regardless of the negative species growth. Other parameter values used are l=1 and (*α*, *β*) are (2, 1) (linear increase); (1, 2) (linear increase); (2, 2) (parabola); and (1, 1) (constant).

**FIGURE A5:**
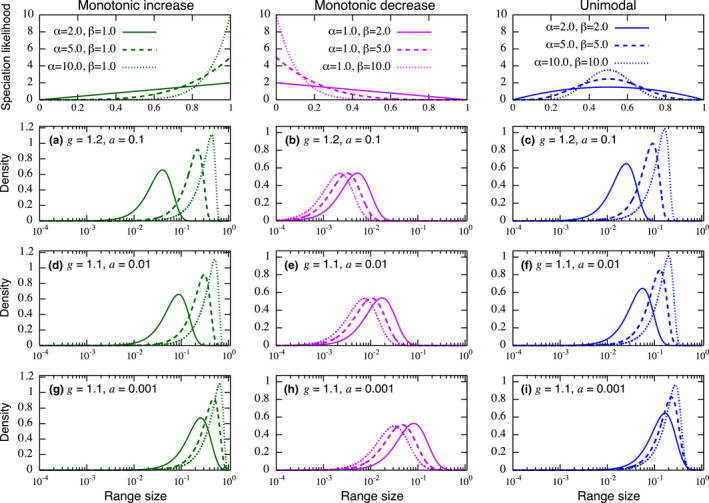
Equilibrium normalized range‐size distributions with nonlinear speciation scenarios: Likelihood of speciation monotonically increases with range size (left), monotonically decreases with range size (center), or peaks at intermediate range size (right). The form of each speciation scenarios is shown in the top panels. Skewness and kurtosis of each equilibrium distribution is provided in Table A1

**FIGURE A6:**
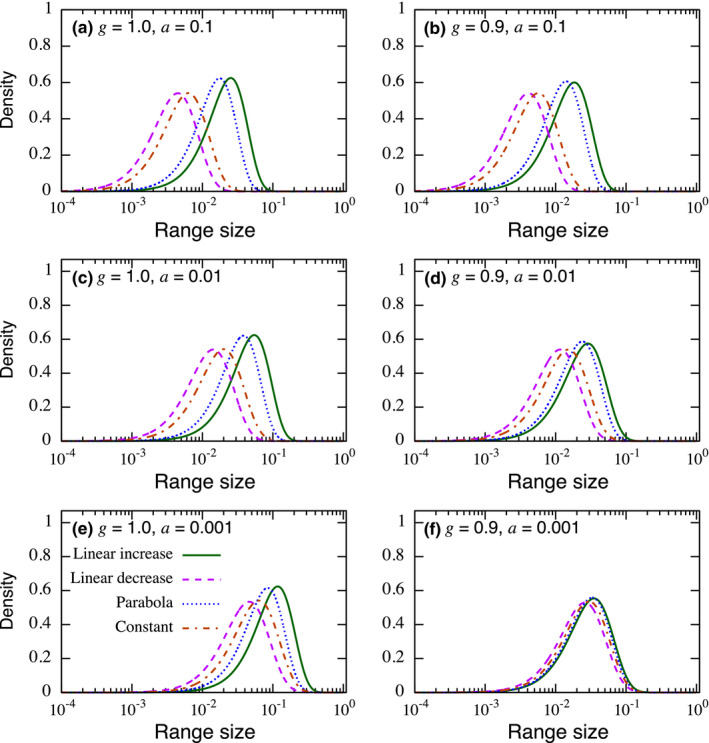
Normalized range‐size distributions in case g=l=1 (left) and g<l=1 (right). All scenarios cause negative growth rate of the number of species. We sampled the range‐size distribution when either the species number becomes 0.01 or range‐size distribution converged to equilibrium regardless of the negative species growth rate. Parameters for the speciation scenarios (*α*, *β*) are (2, 1) (linear increase); (1, 2) (linear increase); (2, 2) (parabola); and (1, 1) (constant)

The gain‐loss‐allopatric speciation (GLAS) model is a general mathematical framework to model the key factors of transformation, speciation, and extinction processes in the dynamics of range‐size distributions. While the components of the model are ultimately phenomenological and not based on modeling the mechanisms driving those processes, it allows us to investigate how their relative rates and functional forms affect range‐size distributions. We found a left‐skewed and lognormal‐like distribution, which has been identified as one of the general patterns in previous studies (Brown et al., 1996; Gaston, 1998; Gaston & Blackburn, 2008; Gaston & He, 2002; Noonan, 1990; Orme et al., 2006), and equivalently a positive skew on a normal axis (Albert et al., 2017; O’Sullivan et al., 2019; Pigot et al., 2010) appears under multiple parameter sets and speciation scenarios (Figure 2, Figures A5 and A6), yet these parameters and speciation scenarios affect the shape statistics of the range‐size distributions (Table 1). Typically, this left‐skewed pattern is observed when the rates of gain and loss of range size are similar in magnitude, and allopatric speciation rates are also of comparable order to these rates. Balanced gain and loss rates avoid a disproportional chance of extinction and an arrival at the large range‐size limit. Allopatric speciation also reduces the chance of a species arriving at the large limit of range size. It induces a split of a range size into two smaller range sizes, pushing the range‐size distribution toward the left side, and we attribute the left‐skew distribution to this mechanism. A lower rate of allopatric speciation allows many species to occupy a larger range (Figure A4). It suggests that the knowledge of the range‐size distribution could be used to diagnose diversification strength relative to the rate of the range‐size transformation. Also, a comparable order of magnitude in the rate of allopatric speciation to the transformation rate is necessary to maintain the diversity. For example, a much larger speciation rate than the transformation rates causes increasingly smaller ranges and it can lead to a larger extinction rate than the speciation rate. On the other hand, biodiversity cannot be maintained with a too small speciation rate due to the lack of a source of new species.

### 4.2 Fitting to range‐size distribution datasets

Our attempts of model fitting produced narrower range‐size distributions than the empirical datasets for mammals, birds, and *Harpalus* carabids. The datasets of *Harpalus* carabids show narrower distributions than the others, and consequently, it leads to a relatively better fit. We hypothesize potential explanations of this variable fitting performance.

First, we assume that all species have the same rate of changes in geographic range size and the speciation rate, and the same speciation scenario. This is a simplification, and wider range‐size distributions of birds and mammals may result in larger heterogeneity in these rates. The number of species is also larger for these datasets than the datasets of *Harpalus* carabids, and these may include more diverse functions, phenotype, evolutionary strategies, and so on, and it leads to more variable parameter values.

Second, we mainly focused on an equilibrium range‐size distribution. However, its transient range size does not yield a unique distribution pattern starting with an arbitrary initial range‐size distribution. In reality, each rate may fluctuate over time and the observed pattern may not be an equilibrium distribution.

Third, environmental factors, for example, occupancy of glaciers (Noonan, 1990), drive transformation of the range sizes. While we assume that the change in range size is based on individual processes where transformation of range size is by one unit, it may not be the case for environmental change where its increments/decrements are proportional to the range size. We provide an example with an assumption of proportional change in range size that leads to wider range‐size distributions in Figure A12 in the Appendix and some technical details below.

### 4.3 Diversification slowdown

**FIGURE A7:**
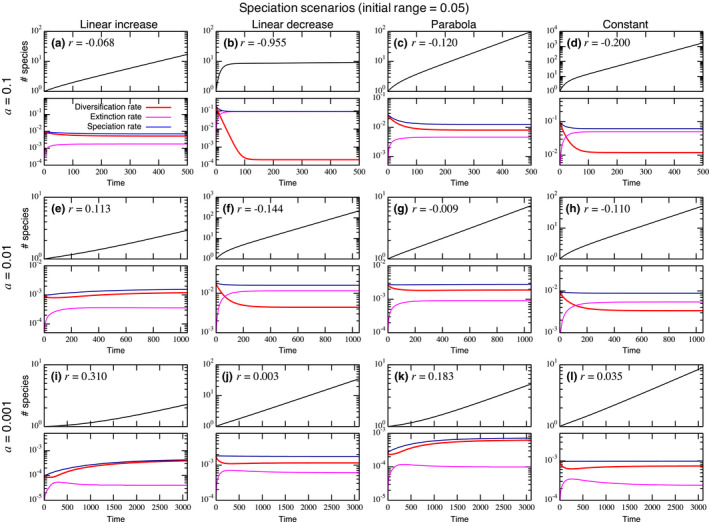
In each panel, (top) lineage‐through‐time plot with the initial condition and (bottom) associated diversification, extinction, and speciation rates. Each simulation is started with a single species with a range size 0.05. Each column shows a different speciation scenario (left to right: linear increase, linear decrease, parabola, and constant), and each row represents a different underlying speciation rate (top: *a* = 0.1; middle: *a* = 0.01; and bottom *a* = 0.001). Other parameter values used are l=1 and g=1.2 for (a)‐(d) and g=1.1 for the rest

**FIGURE A8:**
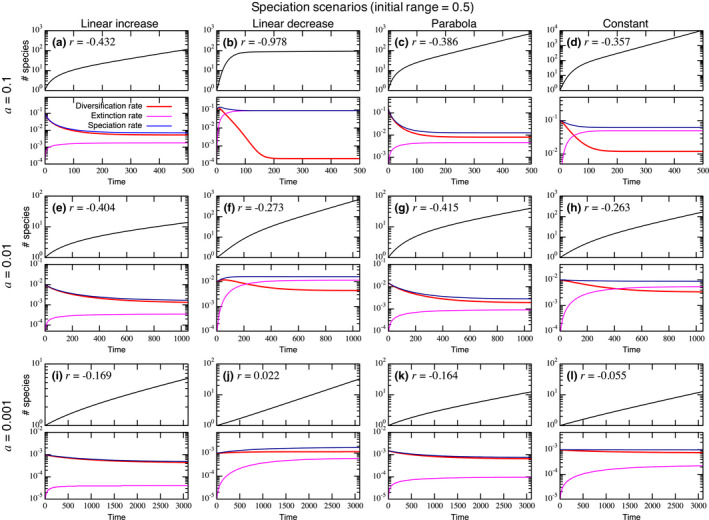
In each panel, (top) lineage‐through‐time plot and (bottom) associated diversification, extinction, and speciation rates. Each simulation is started with a single species with a range size 0.5. Each column shows a different speciation scenario (left to right: linear increase, linear decrease, parabola, and constant), and each row represents a different underlying speciation rate (top: a=0.1; middle: a=0.01; and bottom a=0.001). Other parameter values used are l=1 and g=1.2 for (a)‐(d) and g=1.1 for the rest

**FIGURE A9:**
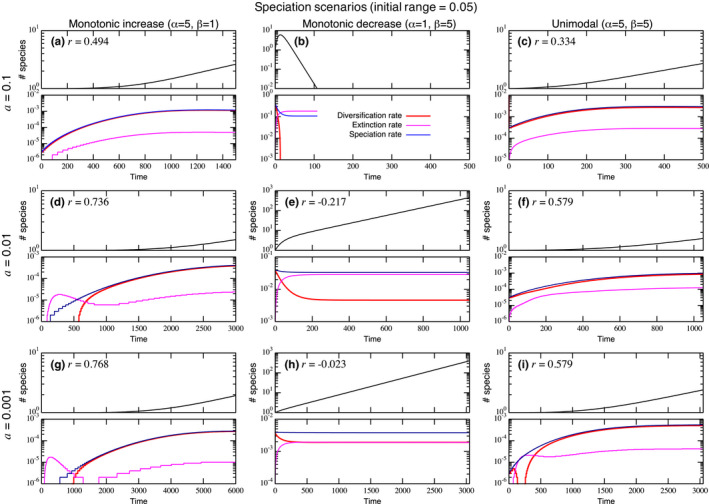
In each panel, (top) lineage‐through‐time plot and (bottom) associated diversification, extinction, and speciation rates. Each simulation is started with a single species with a range size 0.05. Each column shows a different speciation scenario (left to right: monotonic increase, monotonic decrease, unimodal; see Figure A5 top for the form), and each row represents a different underlying speciation rate (top: a=0.1; middle: a=0.01; and bottom a=0.001). Other parameter values used are l=1 and g=1.2 for (a)‐(c) and g=1.1 for the rest. Note the speciation scenario shown in the panel (b) causes all species extinction, and simulation is terminated when the species number becomes 0.01

**FIGURE A10:**
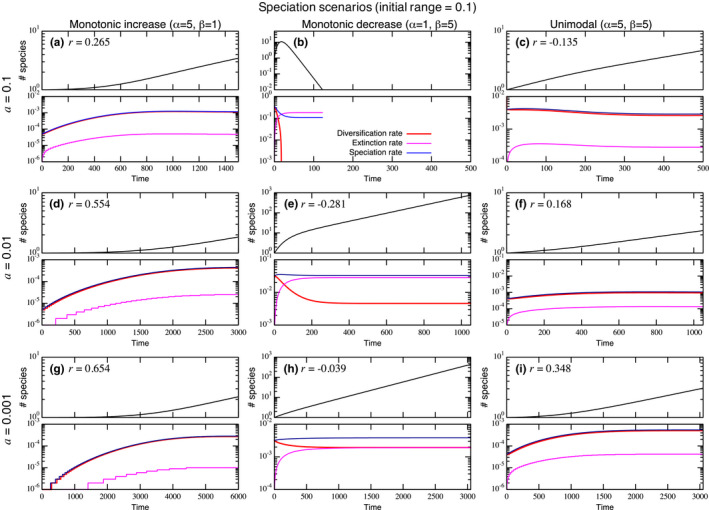
In each panel, (top) lineage‐through‐time plot and (bottom) associated diversification, extinction, and speciation rates. Each simulation is started with a single species with a range size 0.1. Each column shows a different speciation scenario (left to right: monotonic increase, monotonic decrease, unimodal; see Figure A5 top for the form), and each row represents a different underlying speciation rate (top: a=0.1; middle: a=0.01; and bottom a=0.001). Other parameter values used are l=1 and g=1.2 for (a)‐(c) and g=1.1 for the rest. Note the speciation scenario shown in the panel (b) causes all species extinction, and simulation is terminated when the species number becomes 0.01

**FIGURE A11:**
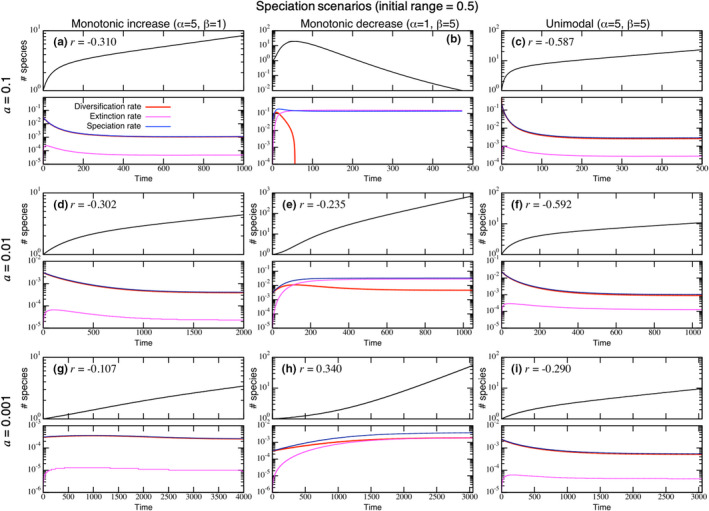
In each panel, (top) lineage‐through‐time plot and (bottom) associated diversification, extinction, and speciation rates. Each simulation is started with a single species with a range size 0.5. Each column shows a different speciation scenario (left to right: monotonic increase, monotonic decrease, unimodal; see Figure A5 top for the form), and each row represents a different underlying speciation rate (top: a=0.1; middle: a=0.01; and bottom a=0.001). Other parameter values used are l=1 and g=1.2 for (a)‐(c) and g=1.1 for the rest. Note the speciation scenario shown in the panel (b) causes all species extinction, and simulation is terminated when the species number becomes 0.01

We show that the GLAS model also produces the signature of diversification slowdown in the phylogeny as in Pigot et al. (2010). In the GLAS model, diversification slowdown occurs during a transient phase as the diversification rate approaches a stable value. Several hypotheses have been discussed to explain these phenomena including density‐dependent speciation (Phillimore & Price, 2008; Rabosky & Lovette, 2008; Weir, 2006), failure to adapt to a changing environment (Quental & Marshall, 2013), and protracted speciation (Etienne & Rosindell, 2012). In our simulations, this slowdown behavior is observed under a wide range of parameter sets and speciation scenarios when the initial range size is closer to a size with high speciation probability. Since speciation tends to move species ranges away from the sizes with highest speciation probability, it tends to push the distribution away as well, slowing down the rate of speciation. For example, when the original species speciates under the linear increase speciation scenario, the two new smaller range sizes may still have a high chance of producing new species before going extinct if the initial species had a relatively large range size. Extinction events are more likely to occur after this transition when there are more species with smaller range sizes, and this eventually suppresses the diversification rate. Although this slowdown is pervasive, opposite trends in the diversification rate can occur (e.g., Figure A7(i) and (k) in Appendix). For example, when the initial species has relatively small range size and speciation probability increases with the range size, there is an initial extinction‐prone period since smaller range sizes are less likely to speciate. After the initial transition period, the speciation rate increases due to the existence of large range sizes, and this leads to an upward curve in the lineage‐through‐time plot. This opposite trend was reported in previous studies and was explained by, for example, climatic cycles and disruptive mountainous terrain where rapid recent speciation might have been induced (Linder et al., 2003; Weir, 2006).

### 4.4 Mechanisms to extend the GLAS model

**FIGURE A12:**
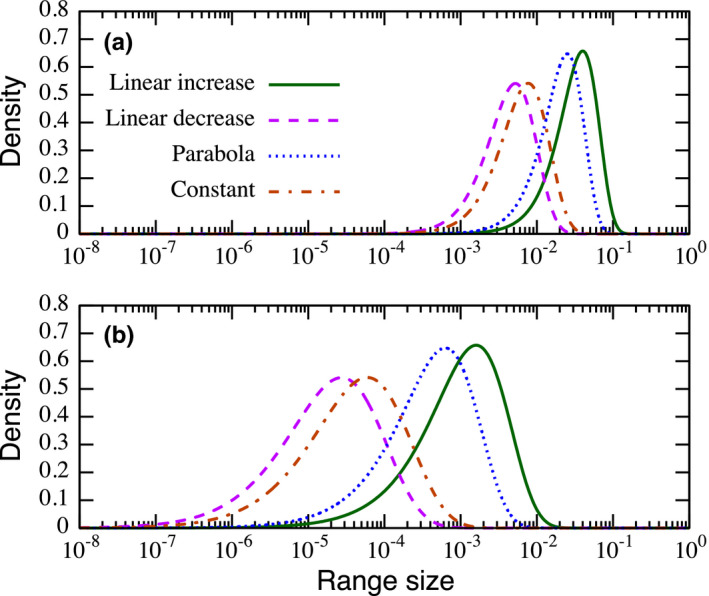
Normalized equilibrium range‐size distributions when the range size *x* (Equation 2) is (a) the range itself; and (b) a diameter of the range (hence $\pi x^{2}$ is the range). Parameter values used are l=1, g=1.2, a=0.1. Also, parameters for the speciation scenarios (*α*, *β*) are (2, 1) (linear increase); (1, 2) (linear increase); (2, 2) (parabola); and (1, 1) (constant)

While our model is a simple approach to modeling the three key factors as discussed in (Gaston, 1998), the results suggest that the model produces widely observed range‐size distributions and diversification slowdown. Thus, it allows us to obtain general insight with minimal assumptions. However, our model can further be extended to investigate a wide range of assumptions, scenarios, and speciation modes. It is possible to assume that the range size *x* in Equation (2) is an arbitrary parameter to characterize a geometry of the range size (e.g., length and perimeter), not necessarily area itself. If the area is a nonlinear function of *x*, then the realized range‐size distribution may have a wider distribution. For example, consider circular ranges, assuming that Equation (2) describes the dynamics of the radius (*x*) of the range, rather than range size ($\pi x^{2}$) itself, Figure A12 shows a wider range‐size distribution under this assumption. This simple example induces the loss of area of the original range size after speciation: If a range size with a radius *x* splits into two ranges with radius *x*_1_ and *x*_2_, then we have $\pi x^{2}\geq \pi \left(x_{1}^{2}+x_{2}^{2}\right)$. While we do not have a clear idea if geographic range size is decreased after allopatric speciation, we can assume various geometries that induces different amounts of area loses after allopatric speciation.

Split asymmetry of ancestral species influences the range‐size evolution (Pigot et al., 2012), and a large asymmetry may also cause a wider range‐size distribution. While our assumption of the equal split probability gives the asymmetric split proportion 25:75, this can be controlled: This effect is incorporated into Equation (2) as $2\int_{x}^{1}a_{2}\left(x^{\prime }\right)a_{x^{\prime }}u\left(x^{\prime },t\right)dx^{\prime }$, where $a_{2}\left(x\right)$ is any symmetric probability distribution function in $0\leq x\leq x_{1}$ with original range size *x*_1_, regulating the degree of the split asymmetry. The average split proportion A:1‐A is obtained from $A=\int_{0}^{x_{1}/2}xa_{2}\left(x\right)dx$. In our analysis, we used $a_{2}\left(x\right)=1/x_{1}$.

We can incorporate range‐size‐dependent gain and loss rates by making them a function of range size g(x) and l(x). It would be possible to relate to population dynamics where, as in Gaston and He (2002), these rates are characterized by colonization and equilibrium phases. Similarly, diversity‐dependent speciation is realized by defining the underlying speciation rate as a function of the total number of species a(N). Other speciation modes such as the point mutation (Hubbell, 2001) are possible to take into account by setting g(0)>0 as in (Volkov et al., 2003). Peripatry may induce larger skew in the range‐size distribution (Pigot et al., 2010), and it can be incorporated by adding a term to Equation (2) that governs the rate of peripatry of a species with a range‐size *x*. Finally, although diversification slowdown is often discussed using reconstructed phylogeny with information extracted from extant species, our discussion is based on the true species number. Developing a method that links to phylogenetic reconstructions would be beneficial for analyzing diversification slowdowns, to directly link to the existing insights.

The shape of species‐range‐size distributions helps us understand the structure of biodiversity across scales. Species‐range‐size distributions are intertwined with other macroecological and community patterns such as species–area and endemic–area relationships and species abundance distributions (Takashina et al., 2019). Thus, further developing our understanding of the dynamics of the range‐size distribution will also promote integrated understanding of many basic and applied macroecological questions.

## CONFLICT OF INTEREST

The authors declare that they have no conflict of interest.

## AUTHOR CONTRIBUTIONS

**Nao Takashina:** Conceptualization (lead); Formal analysis (lead); Funding acquisition (equal); Investigation (lead); Methodology (equal); Project administration (lead); Validation (equal); Writing‐original draft (lead); Writing‐review & editing (equal). **Michael J. Plank:** Conceptualization (supporting); Formal analysis (supporting); Investigation (supporting); Methodology (equal); Validation (equal); Writing‐original draft (supporting); Writing‐review & editing (equal). **Clinton N. Jenkins:** Data curation (lead); Investigation (supporting); Validation (equal); Writing‐review & editing (equal). **Evan P. Economo:** Funding acquisition (equal); Investigation (supporting); Validation (equal); Writing‐original draft (supporting); Writing‐review & editing (equal).

## Data Availability

Species‐range data of mammals and birds in the Americas were from the BirdLife International (BirdLife International & Handbook of the Birds of the World, 2017) and the IUCN Red List (IUCN, 2017) databases. Data of *Harpalus* carabids in North America north of Mexico are available in Noonan (1990).

## APPENDIX 1

### Gain‐Loss‐Allopatric speciation process

Let us assume that each species has a geographic range size with state *r* at time *t* that experiences gain in its range size by one unit (r→r+1) or loss (r→r‐1) at rates *g* and *l*, respectively. In addition, we assume that allopatric speciation can occur at a size‐dependent rate *a_r_*, and it produces two smaller range sizes *r*_1_ and *r*_2_ with one new species so as to satisfy $r_{1}+r_{2}=r$. All combinations of split size are equally likely. These events and probabilities are summarized as follows:

(A1)
$$r\to \left\{\begin{array}{cc} r+1, & g\Delta t\\ r, & 1‐\left(g+l+a_{r}\right)\Delta t\\ r‐1, & l\Delta t\\ r‐1,1, & \frac{a_{r}}{r‐1}\Delta t\\ r‐2,2, & \frac{a_{r}}{r‐1}\Delta t\\ \vdots & \\ 2,r‐2, & \frac{a_{r}}{r‐1}\Delta t\\ 1,r‐1, & \frac{a_{r}}{r‐1}\Delta t \end{array}\right.$$

We define an extinction as the event that a species achieves range size 0, which can only occur from *r* = 1. These events are translated into the following stochastic process that describes the dynamics of the expected number of species with range size *r* at time *t*:

(A2)
$$\frac{dP(r,t)}{\mathrm{dt}}=gP\left(r‐1,t\right)+lP\left(r+1,t\right)+2\sum_{r^{\prime }>r}\frac{a_{r^{\prime }}}{r^{\prime }‐1}P\left(r^{\prime },t\right)‐\left(g+l+a_{r}\right)P\left(r,t\right),$$

where *g* and *l* are the gain and loss rate of geographic range, and *a_r_* are the size‐dependent allopatric speciation rate. Here, we perform a continuum limit of Equation (1).

### Some dynamical properties of the GLAS process

Since Equation (A2) is linear, we can rewrite the equation with a matrix form

(A3)
$$\frac{\mathrm{dP}}{\mathrm{dt}}=MP,$$

where $P=(P(1,t),P(2,t),\cdots ,P(r_{\max},t)){\;}^{T}$ and *M* is the coefficient matrix of Equation (A2). Therefore, the real part of the dominant eigenvalue Re(λmax) determines if the *expected* species number described by the GLAS process in the long time limit exponentially increases (Re(λmax)>0), exponentially decreases (Re(λmax)<0), or has a stable number of species (Re(λmax)=0). Figure A1 shows such values. The curve in each panel corresponds to the Re(λmax)=0, and the upper and lower regions are Re(λmax)>0 and Re(λmax)<0, respectively.

## APPENDIX 2

### Continuum limit of the glas process

It is reasonable to consider that the geographic range size changes continuously rather than a discrete manner. The continuum limit of the GLAS process is expected to inherit dynamical property of the GLAS process mentioned above. In the following, we will use a standard procedure to obtain such a model (see, e.g., Gardiner, 2009).

First, let Δx be a small step size, and let x=rΔx. Then, we define the new variable u(x,t) where the number of species between range size *x* and x+Δx at time *t* is u(x,t)Δx. By expanding u(x,t) to the second order of Δx, we have the following integro‐differential equation:

(A4)
$$\begin{array}{cc} \frac{\partial u\left(x,t\right)}{\partial t} & =gu\left(x‐\Delta x,t\right)+lu\left(x+\Delta x,t\right)+2\int_{x}^{x_{\max}}\frac{a_{x^{\prime }}}{x^{\prime }}u\left(x^{\prime },t\right)dx^{\prime }‐\left(g+l+a_{x}\right)u\left(x,t\right),\\ & =‐V\frac{\partial u\left(x,t\right)}{\partial x}+D\frac{\partial^{2}u\left(x,t\right)}{\partial x^{2}}+2\int_{x}^{x_{\max}}\frac{a_{x^{\prime }}}{x^{\prime }}u\left(x^{\prime },t\right)dx^{\prime }‐a_{x}u\left(x,t\right), \end{array}$$

where V=(g‐l)Δx and $D=\frac{g+l}{2}\Delta x^{2}$, and we replace $\Sigma_{r^{\prime }>r}$ with $\int_{x}^{x_{\max}}$ and $a_{x}$ is the size‐dependent split rate.

As in the main text, let us normalize the range size x∈[0,1], and if we assume that a loss of species only occurs when the range size of a species achieves x=0. To guarantee this condition, we require a Robin boundary condition at the upper boundary and we have the following mixed boundary conditions:

(A5)
$$\left\{\begin{array}{c} u\left(0,t\right)=0\\ ‐Vu\left(1,t\right)+Du^{\prime }\left(1,t\right)=0 \end{array}\right.$$

where $u^{\prime }$ indicates the derivative with respect to *x*.

### Dynamics of total species

The dynamics of the total number of species $N\left(t\right)=\int_{0}^{1}u\left(x,t\right)dx$ is derived by integrating both sides of Equation (A4) with respect to *x* to obtain

$$\begin{array}{cc} \frac{dN\left(t\right)}{\mathrm{dt}} & =‐Vu\left(1,t\right)+D\left(u^{\prime }\left(1,t\right)‐u^{\prime }\left(0,t\right)\right)+\int_{0}^{1}\left[2\int_{x}^{1}\frac{a_{x^{\prime }}}{x^{\prime }}u\left(x^{\prime },t\right)dx^{\prime }‐a_{x}u\left(x,t\right)\right]dx,\\ & =‐Du^{\prime }\left(0,t\right)+\int_{0}^{1}\left[2\int_{x}^{1}\frac{a_{x^{\prime }}}{x^{\prime }}u\left(x^{\prime },t\right)dx^{\prime }‐a_{x}u\left(x,t\right)\right]dx, \end{array}$$

where we have used the condition $‐Vu\left(1,t\right)+Du^{\prime }\left(1,t\right)=0$. Interchanging the order of the double integral, this becomes

(A6)
$$\frac{dN\left(t\right)}{\mathrm{dt}}=‐Du^{\prime }\left(0,t\right)+\int_{0}^{1}a_{x}u\left(x,t\right)dx.$$

The first term of the right‐hand side of Equation (A6) corresponds to species loss due to extinction, and the second term represents species increment due to speciation events. Per‐species rates of extinction and speciation events are obtained by dividing by the total number of species at given time N(t).

Using Equation (A6), the diversification rate at time *t* can be computed. Diversification rate at given time λ(t) is the rate of increase in the log‐number of species dlogN(t)/dt and has the following relationship: λ(t)=speciationrateperspeciesattimet‐extinctionrateperspeciesattimet. It is expected that the diversification rate asymptotically converges to the dominant eigenvalue $\lambda_{\max}=\lim_{t\to \infty }\left(\log N(t)/t\right)$ of the coefficient matrix in Equation (A3) as *t* becomes sufficiently large.

### Solution of Equation (A4) in a special case

It is possible to derive an analytical solution when the speciation scenario is linear increase in a special situation. Although the assumptions allow us to analyze restricted situations, this derivation could help further analytical development of the GLAS process with more general assumptions.

Overall, the following assumptions are made for make the analysis of the Laplace‐transformed integration term possible. First, we assume the boundary condition at the upper limit is either Dirichlet boundary condition $u(x_{\max},t)=0$ or bounded at infinity $\lim_{x\to \infty }u\left(x,t\right)=0$. As an example, we will use the Diriclet boundary condition in the following. Next, we transform the dynamics of species number into fraction of species. To do this, let $\tilde{u}\left(x,t\right)\Delta x$ be the fraction of species that range size is between *x* and x+Δx, namely

(A7)
$$\tilde{u}\left(x,t\right)\Delta x=\frac{u\left(x,t\right)\Delta x}{\int_{0}^{x_{\max}}u\left(x,t\right)dx}=\frac{u\left(x,t\right)\Delta x}{N\left(t\right)},$$

and then $\partial u/\partial t=\partial \tilde{u}/\partial tN+\tilde{u}dN/dt$. Therefore, when 1/NdN/dt is sufficiently small, this changes in variable holds the same form as Equation (A4), and we assume this situation. Combined with these assumptions and the speciation scenario of linear increase ($a_{x}=2ax$), we have the followings

(A8)
$$\left\{\begin{array}{c} \frac{\partial \tilde{u}\left(x,t\right)}{\partial t}=‐V\frac{\partial \tilde{u}\left(x,t\right)}{\partial x}+D\frac{\partial^{2}\tilde{u}\left(x,t\right)}{\partial x^{2}}+4a\int_{x}^{x_{\max}}\tilde{u}\left(x^{\prime },t\right)dx^{\prime }‐a\tilde{u}\left(x,t\right),\\ \tilde{u}\left(0,t\right)=0,\tilde{u}\left(x_{\max},t\right)=0\\ \tilde{u}\left(x,0\right)=\delta_{0}\left(x_{0}\right),\;x_{0}\in \left(0,x_{\max}\right) \end{array}\right.$$

By Laplace transforming $L\tilde{u}\left(x,t\right)=\int_{0}^{\infty }\tilde{u}\left(x,t\right)e^{‐sx}dx=\tilde{U}\left(s,t\right)$ both side in terms of *x*, we get

(A9)
$$\begin{array}{cc} \frac{\partial \tilde{U}\left(s,t\right)}{\partial t} & =‐V\left(s\tilde{U}\left(s,t\right)‐\tilde{u}\left(0,t\right)\right)+D\left(s^{2}\tilde{U}\left(s,t\right)‐s\tilde{u}\left(0,t\right)‐{\tilde{u}}^{\prime }\left(0,t\right)\right)\\ & \;+4a\left(\frac{1}{s}‐\frac{\tilde{U}\left(s,t\right)}{s}\right)‐a\tilde{U}\left(s,t\right),\\ & =\tilde{U}\left(s,t\right)\underset{A}{\underbrace{\left(‐Vs+Ds^{2}‐\frac{4a}{s}‐a\right)}}‐D{\tilde{u}}^{\prime }\left(0,t\right)+\frac{4a}{s}, \end{array}$$

where the Laplace transformation of integration term is obtained by the integration by parts and used the above‐mentioned assumptions $\int_{0}^{x_{\max}}\tilde{u}\left(x,t\right)dx=1$ and $\tilde{u}\left(x_{\max},t\right)=0$. Equation (A9) is actually a first‐order linear ordinary differential equation, and hence, it is easy to solve. With an initial condition that range size of all species is concentrated at a single value $\tilde{u}\left(x,0\right)=\delta_{0}\left(x_{0}\right)$ and $\tilde{U}\left(s,0\right)=1$, we obtain the following solution

(A10)
$$\tilde{U}\left(s,t\right)=‐\frac{4a}{\mathrm{As}}\left(1‐e^{\mathrm{At}}\right)+e^{\mathrm{At}},$$

To perform inverse Laplace transform, we further need to arrange Equation (A10):

(A11)
$$\begin{array}{cc} \tilde{U}\left(s,t\right) & =1+\left(‐Vs+Ds^{2}‐\frac{8a}{s}‐a\right)\left(t+\frac{t^{2}A}{2!}+\frac{t^{3}A^{2}}{3!}+\cdots \right),\\ & =1+\left(‐Vs+Ds^{2}‐\frac{8a}{s}‐a\right)\sum_{n=1}^{\infty }\frac{t^{n}}{n!}\times \\ & \;\sum_{\left|k\right|=n‐1}\left(\begin{array}{c} n‐1\\ k \end{array}\right)(‐Vs){\;}^{k_{1}}(Ds^{2}){\;}^{k_{2}}{\left(‐\frac{4a}{s}\right)}^{k_{3}}(‐a){\;}^{k_{4}},\\ & =1+\sum_{n=1}^{\infty }\sum_{\left|k\right|=n‐1}\frac{t^{n}\left(‐Vs+Ds^{2}‐\frac{8a}{s}‐a\right)}{nk!}(‐Vs){\;}^{k_{1}}(Ds^{2}){\;}^{k_{2}}{\left(‐\frac{4a}{s}\right)}^{k_{3}}(‐a){\;}^{k_{4}}, \end{array}$$

where we used multi‐index notations $\left|k\right|=k_{1}+k_{2}+k_{3}+k_{4}$, $\left(\begin{array}{c} n\\ k \end{array}\right)=n!/\left(k_{1}!k_{2}!k_{3}!k_{4}!\right)$ and $k!=k_{1}!k_{2}!k_{3}!k_{4}!$. To derive the second line, we applied the multinomial theorem to *A*. Equation (A10) is now the form to be able to inverse Laplace transform. By applying the inverse Laplace operator to both side, and calculate it separately for each term contains *s*, we get the final result

(A12)
$$\begin{array}{cc} \tilde{u}\left(x,t\right) & =\delta_{0}\left(x_{0}\right)+\sum_{n=1}^{\infty }\sum_{\left|k\right|=n‐1}\frac{t^{n}x^{‐k_{1}‐2k_{2}+k_{3}‐3}}{nk!}{\left(‐1\right)}^{k_{3}+k_{4}}2^{3k_{3}+k_{4}}{\left(‐V\right)}^{k_{1}}D^{k_{2}}\times \\ & \left\{\frac{D}{\Gamma \left(‐k_{1}‐2k_{2}+k_{3}‐2\right)}‐\frac{\mathrm{Vx}}{\Gamma \left(‐k_{1}‐2k_{2}+k_{3}‐1\right)}+\frac{2x^{2}\left(k_{1}+2k_{2}‐k_{3}‐8x\right)}{\Gamma \left(‐k_{1}‐2k_{2}+k_{3}+1\right)}\right\}, \end{array}$$

where Γ(n) is the gamma function.

### Logarithmic transformation of Equation (A4)

Since datasets of species‐range‐size distribution often show a diverse variations of range sizes on the logarithmic axis, it is convenient to employ a log‐transformed model in model fitting where range sizes are equally spaced on a logarithmic axis. The logarithmic transformation of the GLAS process is achieved by introducing a new variable $y=\ln x/x_{0}$ where $x_{0}$ is the reference point. Under this transformation, we describe the number of species between *y* and y+Δy at time *t* by v(y,t)Δy, and replacing u(x,t) in Equation (A4) with v(y,t) provides the log‐transformed GLAS process:

(A13)
$$\begin{array}{cc} \frac{\partial v\left(y,t\right)}{\partial t} & =‐\left(V\frac{e^{‐y}}{x_{0}}+D\frac{e^{‐2y}}{x_{0}^{2}}\right)\frac{\partial v\left(y,t\right)}{\partial y}+D\frac{e^{‐2y}}{x_{0}^{2}}\frac{\partial^{2}v\left(y,t\right)}{\partial y^{2}}\\ & +2\int_{y}^{\ln1/x_{0}}a_{y}^{\prime }v\left(y^{\prime },t\right)dy^{\prime }‐a_{y}v\left(y,t\right), \end{array}$$

where the boundary condition as Equation (A5) is

(A14)
$$\left\{\begin{array}{c} v\left(0,t\right)=0,\\ \left(V\frac{e^{‐y}}{x_{0}}+D\frac{e^{‐2y}}{x_{0}^{2}}\right)v\left(1,t\right)‐D\frac{e^{‐2y}}{x_{0}^{2}}\frac{\partial v\left(1,t\right)}{\partial y}=0. \end{array}\right.$$

## APPENDIX 3

### Technical details of the optimization algorithm

We discuss here the settings of simulated annealing (Kirkpatrick et al., 1983) used in data fitting. This is a heuristic searching process of local optima of an objective function f(S) until the temperature, T, becomes lower than predetermined minimum temperature $T_{\min}$ or an objective function (a log‐likelihood) meets the predetermined target. We provide pseudocode as follows:

In our case, there are four parameters to optimize (g,s,α,β) and these are chosen randomly at each step for updating the value. We heuristically define parameters in the algorithm that induce convergence trends in the optimization $\left(T,T_{\min},Target,c\right)=\left(10^{2},10^{‐5},‐1,0.99\right)$. For a random value ε on the line 4, we used a normal distribution with mean 0 and parameter‐dependent variance $\left(\sigma_{g}^{2},\sigma_{s}^{2},\sigma_{\alpha }^{2},\sigma_{\beta }^{2}\right)=\left(5\times 10^{‐2},10^{‐3},0.5,0.5\right)$, where the subscripts indicate a corresponding parameter. A on the Line 5 is a random variable drawn from the uniform distribution with the range [0,1]. We drawn initial parameters from the uniform distributions: g∈U[1.1,1.6], s∈U[0.001,1] and α,β∈U[1,50], where we used a uniform distribution on a log‐axis for *s*.

**TABLE A1:** Summary of (skewness, kurtosis) from the each curve in Figure A5

(*α*, *β*)	*a* = 10^−1^	*a* = 10^−2^	*a* = 10^−3^
*Monotonic increase*			
(2,1)	(−1.016,1.913)	(−1.020,1.926)	(−1.045,2.013)
(5,1)	(−1.497,3.645)	(−1.488,3.616)	(−1.500,3.656)
(10,1)	(−1.702,4.413)	(−1.697,4.399)	(−1.701,4.414)
*Monotonic decrease*			
(1,2)	(−0.777,1.182)	(−0.771,1.170)	(−0.732, 1.100)
(1,5)	(−0.774,1.175)	(−0.759, 1.146)	(−0.674, 1.002)
(1,10)	(−0.770,1.168)	(−0.747, 1.123)	(−0.612, 0.907)
*Unimodal*			
(2,2)	(−0.996, 1.847)	(−0.989, 1.832)	(−0.953, 1.783)
(5,5)	(−1.425, 3.394)	(−1.383, 3.249)	(−1.300, 3.022)
(10,10)	(−1.641, 4.202)	(−1.605, 4.077)	(−1.524, 3.829)
